# Critical evidence for the prediction error theory in associative learning

**DOI:** 10.1038/srep08929

**Published:** 2015-03-10

**Authors:** Kanta Terao, Yukihisa Matsumoto, Makoto Mizunami

**Affiliations:** 1 Graduate School of Life Science, Hokkaido University, Sapporo 060-0810, Japan; 2 Faculty of Science, Hokkaido University, Sapporo 060-0810, Japan; 3 Faculty of Liberal Arts, Tokyo Medical and Dental University, Ichikawa 272-0827, Japan

## Abstract

In associative learning in mammals, it is widely accepted that the discrepancy, or error, between actual and predicted reward determines whether learning occurs. Complete evidence for the prediction error theory, however, has not been obtained in any learning systems: Prediction error theory stems from the finding of a blocking phenomenon, but blocking can also be accounted for by other theories, such as the attentional theory. We demonstrated blocking in classical conditioning in crickets and obtained evidence to reject the attentional theory. To obtain further evidence supporting the prediction error theory and rejecting alternative theories, we constructed a neural model to match the prediction error theory, by modifying our previous model of learning in crickets, and we tested a prediction from the model: the model predicts that pharmacological intervention of octopaminergic transmission during appetitive conditioning impairs learning but not formation of reward prediction itself, and it thus predicts no learning in subsequent training. We observed such an “auto-blocking”, which could be accounted for by the prediction error theory but not by other competitive theories to account for blocking. This study unambiguously demonstrates validity of the prediction error theory in associative learning.

Understanding computational rules underlying associative learning, such as classical conditioning and operant conditioning, is a major goal of neuroscience. In associative learning in mammals, it is widely accepted that the discrepancy, or error, between the actual unconditioned stimulus (US) and the predicted US determines whether learning occurs when a stimulus is paired with the US^1^. This theory stems from the finding of “blocking” by Kamin^2^. He observed, in rats, that a stimulus X that had been paired previously with a US could block subsequent association of a second stimulus Y to the US when the two stimuli were paired in compound with the same US. Kamin^2^ argued that the blocking is due to the requirement of surprise for learning, i.e., no learning occurs when the US is fully predicted, and this proposition was formulated into the prediction error theory by Rescorla and Wagner^3^. Recent neuroscience research in mammals has demonstrated that activities of dopamine (DA) neurons in the ventral tegmental area of the midbrain mediate prediction error signals in classical conditioning^1^,^4^ and instrumental conditioning^5^.

Unambiguous demonstration of the prediction error theory, however, has not been achieved in any learning systems. Blocking can also be accounted for by theories other than the prediction error theory^6^,^7^,^8^, and thus experiments are needed to discriminate among different theories. The most influential theory is the attentional theory (or theory of attention) proposed by Machintosh^9^ and Pearce and Hall^10^, which accounts for blocking by a loss of attention to a stimulus. Another notable theory is the comparator hypothesis^11^, which accounts for blocking by cue competition during memory retrieval. Experiments have been performed to discriminate the prediction error theory from other theories in some learning systems^6^,^7^,^8^, but unequivocal evidence to reject all alternative theories has not been obtained in any learning systems.

Studies in invertebrates have greatly contributed to an understanding of cellular and molecular mechanisms of associative learning^12^,^13^,^14^,^15^,^16^, but whether the prediction error theory is applicable to invertebrates has not been examined. One of the reasons for the lack of such study is the difficulty in establishing experimental procedures to convincingly demonstrate blocking. In insects, for example, earlier studies in honey bees showed a blocking-like effect in free-flight stimulus selection experiments^17^,^18^ and in classical conditioning of proboscis extension responses^19^,^20^,^21^, but subsequent studies have failed to establish blocking as a robust learning phenomenon^22^,^23^,^24^. Another reason is that, even though blocking has been established in some invertebrates, especially mollusks^25^,^26^,^27^,^28^, experiments have not been performed to discriminate different theories of blocking in any invertebrate species.

In this study, we obtained unequivocal evidence of blocking in classical conditioning in crickets. Crickets are newly emerging experimental animals, in which neural mechanisms of classical conditioning have been unraveled in some detail^29^. For example, we observed that octopamine (OA) receptor antagonists impair appetitive learning but not aversive learning, whereas DA receptor antagonists impair aversive learning but not appetitive learning^30^,^31^,^32^. Moreover, we observed that OA receptor antagonists impair retrieval of appetitive memory but not that of aversive memory, whereas DA receptor antagonists impair retrieval of aversive memory but not that of appetitive memory^33^. Therefore, we concluded that OA neurons and DA neurons code appetitive and aversive US in conditioning and that activation of these neurons is also needed for retrieval of appetitive and aversive memory, respectively. We also proposed a neural model of classical conditioning to account for these finindgs^33^ and showed that the model accounts for some of higher-order learning phenomena such as second-order conditioning^33^ and sensory preconditioning^34^. In this study, we performed behavioral analysis of blocking in crickets and obtained evidence to support the prediction error theory. Moreover, we proposed a neural model that matches the prediction error theory by modifying our previous model and performed pharmacological tests of a prediction from the model. The results unambiguously demonstrated applicability of the prediction error theory to classical conditioning in crickets.

## Results

### Effects of compound conditioning

**Figure 1:**
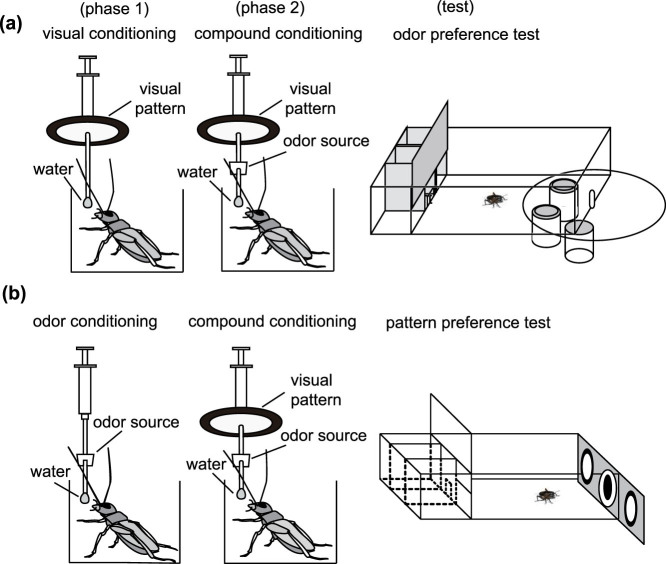
Procedures for blocking experiments. (a) Procedures for the study of blocking of olfactory learning in crickets. A group of water-deprived crickets, individually placed in a beaker, was subjected to pairings of a visual pattern with water US (P+ training) as phase 1 training, and then pairings of an odor-pattern compound with water US (OP+ training) as phase 2 training. Relative preference for the conditioned odor and a control odor was tested before and after training in a test chamber. (b) Procedures for the study of blocking of visual pattern learning. Another group of crickets was subjected to pairings of an odor with water US (O+ training) and then pairings of an odor-pattern compound with water (OP+ training). Relative preference for the conditioned pattern and a control pattern was tested before and after training in a test chamber.

**Figure 2:**
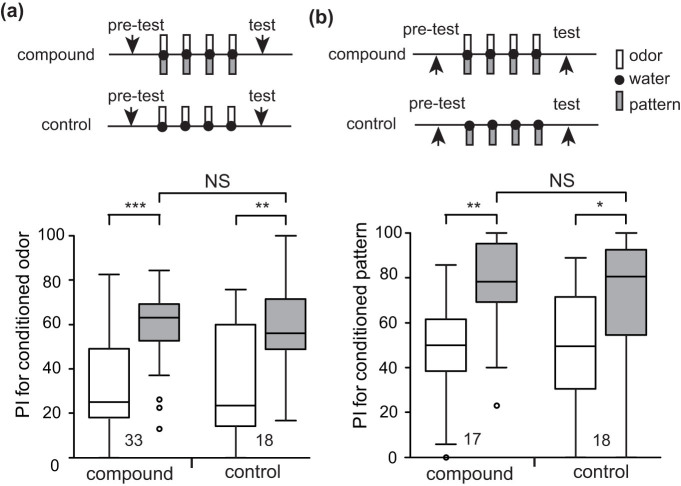
Effect of odor-pattern compound conditioning. (a) Olfactory learning by odor-pattern compound conditioning. One group of animals (compound group) was subjected to 4-trial pairing of an odor-pattern compound with water US and another group (control group) was subjected to 4-trial pairing of an odor alone with water US. (b) Visual learning by odor-pattern compound conditioning. One group was subjected to 4-trial pairing of an odor-pattern compound with water and another group was subjected to 4-trial pairing of a pattern with water. The inter-trial interval (ITI) was 5 min. Relative preference for conditioned odor and control odor or for conditioned pattern and control pattern was tested before and at 30 min after training. The experimental procedures are illustrated at the top, and preference indexes (PIs) for the rewarded odor or pattern before (white bars) and after (grey bars) training are shown as box and whisker diagrams at the bottom. The horizontal line in the box is the median and the box represents the 25–75 percentiles in this and in all following figures. Whiskers extend to extreme values as long as they are within a range of 1.5 × box length. The outliers are shown as open circles. The number of animals is shown below the boxes. The WCX test and the M-W test were used for comparison of preferences before and after conditioning and preferences between groups, respectively. For multiple comparisons, Holm's method was used to adjust the significance level. The results of statistical comparison are shown as asterisks (** p < 0.01; * p < 0.05; NS p > 0.05).

In experiments to study blocking, we used odor-pattern compound conditioning (OP+ conditioning), in which a compound stimulus consisting of an odor (O) and a visual pattern (P) is paired with a water US (reward) (+) (Table 1). The procedures for experiments are described in the Methods section and illustrated as parts of Fig. 1. As a prerequisite for such experiments, we first tested whether OP+ training leads to learning of both the odor and the visual pattern. One group of animals was subjected to 4-trial OP+ training and another group (control group) was subjected to 4-trial olfactory conditioning (O+ conditioning). In both groups, the relative preference for the conditioned odor and control odor was tested before and at 30 min after training. The OP+ group exhibited a significantly greater preference for the odor after training than that before training (W = 23, p = 0.00000015, WCX test) as did the O+ group (W = 17, p = 0.0016, WCX test; Fig. 2a). Between-group comparison showed that there was no significant difference in preference for the odor after training between the OP+ and O+ groups (W = 118.5, p = 0.79, M-W test). Thus, we conclude that odor-pattern compound conditioning leads to conditioning of the odor.

Next, one group of animals was subjected to 4-trial OP+ training and another group (control group) was subjected to 4-trial visual pattern conditioning (P+ training). The OP+ group exhibited a significantly greater preference for the pattern after training than that before training (W = 15, p = 0.0021, WCX test) as did the P+ group (W = 32, p = 0.018; Fig. 2b). Between-group comparison showed that the preference for the pattern after training of the OP+ group did not significantly differ from that of the P+ group (U = 142, p = 0.73, M-W test). Thus, odor-pattern conditioning also leads to conditioning of the pattern.

### Demonstration of blocking

**Figure 3:**
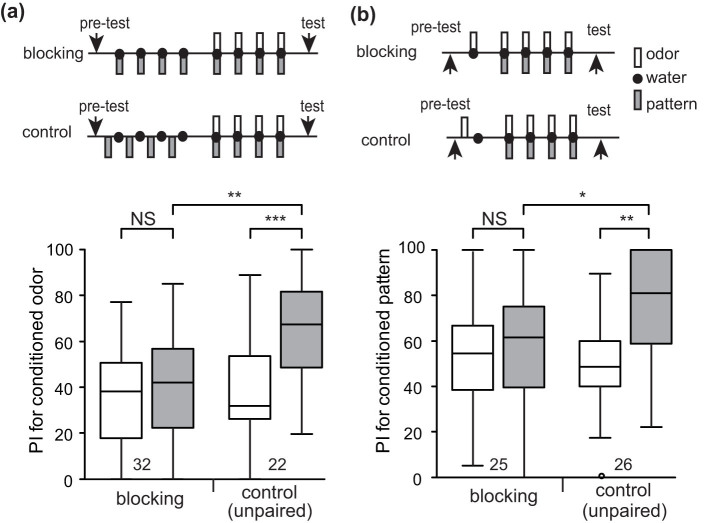
Blocking of visual and olfactory learning. (a) Blocking of olfactory learning. One group of animals (blocking group) was subjected to 4-trial pairing of a pattern with water, and 30 min later the group was subjected to 4-trial pairing of an odor-pattern compound with water US. Another group (unpaired group) was subjected to unpaired presentation of a pattern and water 4 times each, and 30 min later the group was subjected to 4-trial pairing of an odor-pattern compound with water. (b) Blocking of visual learning. One group was subjected to 1-trial pairing of an odor with water and 30 min later subjected to 4-tiral pairing of an odor-pattern compound with water. Another group was subjected to unpaired presentation of an odor and water and 30 min later subjected to 4-trial pairing of an odor-pattern with water. The ITI was 5 min. Relative preference for odors or patterns was tested before and at 30 min after training. PIs for the rewarded odor or pattern before (white bars) and after (gray bars) training are shown as box and whisker diagrams. The number of animals is shown below the boxes. The WCX test was used for comparison of preferences before and after conditioning, and the M-W test was used to compare between groups. For multiple comparisons, Holm's method was used to adjust the significance level. The results of statistical comparison are shown as asterisks (*** p < 0.001; * p < 0.05; NS p > 0.05).

We next studied whether blocking occurs in crickets. One group of animals (blocking group) was subjected to 4-trial P+ training and then 4-trial OP+ training (Table 1). Another group (unpaired group) was subjected to unpaired presentations of a visual pattern and reward (P/+) 4 times each and then 4-trial OP+ training. The blocking group exhibited no significantly increased preference for the odor after training than that before training (W = 169, p = 0.077, WCX test; Fig. 3a). In contrast, the unpaired group exhibited significantly increased preference for the odor after training than that before training (W = 20, p = 0.00018, WCX test). Between-group comparison showed that the preference for the odor after training was significantly less in the blocking group than that in the unpaired group (U = 537.5, p = 0.0011, M-W test; Fig. 3a) or in the compound group (U = 770, p = 0.0015, M-W test; Fig. 2A). The results demonstrate blocking of learning of an odor.

Similarly, we observed blocking of visual pattern learning. One group of animals was subjected to 1-trial O+ training and then 4-trial OP+ training (Fig. 3b). In this experiment, 1-trial O+ training was sufficient because O+ training is more effective than P+ training^30^,^31^. Another group was subjected to unpaired presentations of an odor and reward (O/+) once and then 4-trial OP+ training. The blocking group exhibited no significantly increased preference for the pattern after training than that before training (W = 134, p = 0.46, WCX test). In contrast, the unpaired group exhibited significantly increased preference for the pattern after training than that before training (W = 48, p = 0.00066, WCX test). Between-group comparison showed that the preference for the pattern after training was significantly less in the blocking group than that in the unpaired group (U = 201, p = 0.020, M-W test; Fig. 3b) or the compound group (U = 309.5, p = 0.013, M-W test; Fig. 2b).

### Evaluation of the attentional theory: demonstration of blocking with 1-trial compound conditioning

**Figure 4:**
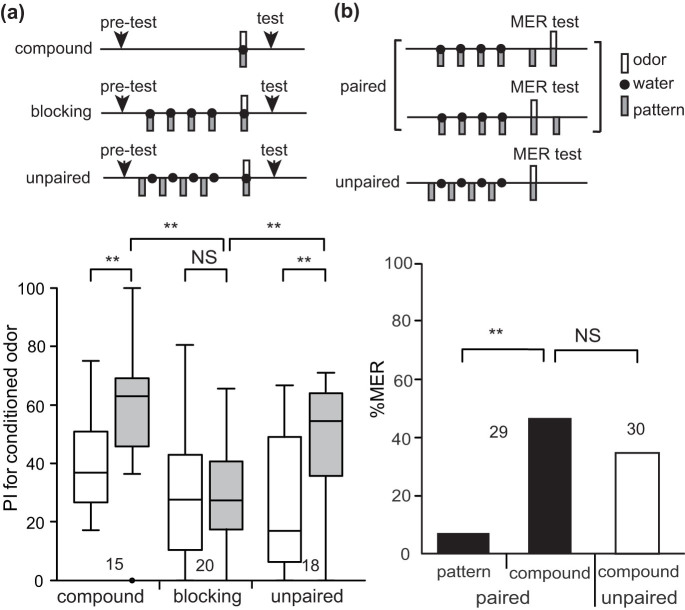
Experiments to discriminate the prediction error theory and attentional theory. (a) Blocking with 1-trial compound conditioning. One group of animals (compound group) was subjected to 1-trial pairing of an odor-pattern compound with water. Another group (blocking group) was subjected to 4-trial pairing of a pattern with water, and 30 min later subjected to 1-trial pairing of an odor-pattern compound with water. Another group (unpaired group) was subjected to unpaired presentation of an odor-pattern compound and water 4 times each, and 30 min later subjected to 1-trial pairing of an odor-pattern compound with water. The ITI was 5 min. Relative odor preferences were tested before and at 30 min after training. PIs for the rewarded odor before and after conditioning are shown as box and whisker diagrams. The WCX test was used for comparison of preferences before and after conditioning, and the M-W test was used to compare between groups. (b) Test of attention to an odor presented in odor-pattern compound after pattern conditioning. Two groups of animals were subjected to 4-trial pairing of a pattern with water, and 30 min later one group was tested with MER to the pattern and then to an odor-pattern compound and another group was tested with reversed sequences. Data from the two groups were pooled to measure percentage of MER to the pattern and the compound stimuli because the sequence of tests had no effect. Another group was subjected to unpaired presentation of a pattern and water 4 times each, and 30 min later an odor-pattern compound was presented to test MER. The ITI in phase 1 training was 5 min and the interval between phase 1 and phase 2 training was 30 min. Bars represent percentage of MER to the CS. The number of animals is shown in the figure. McNemar's test was used for comparison of %MER to pattern and compound stimuli. Fisher's exact test was used to compare between groups. For multiple comparisons, Holm's method was used to adjust the significance level. The results of statistical comparison are shown as asterisks (** p < 0.01; NS p > 0.05).

Prediction error theory is a predominant theory to account for blocking^7^,^8^, but a few other theories can also explain blocking. The most influential one is attentional theory, which accounts for blocking by a loss of attention to a stimulus^9^,^10^. We investigated which theory, the attentional theory or the prediction error theory, better accounts for blocking in crickets; other theories will be discussed later. The decisive method to discriminate these two theories is to study the effect of blocking with X+ training and subsequent 1-trial XY+ training: the attentional theory predicts a loss of attention to Y after the first XY+ training (not after X+ training), and thus 2-trial XY+ training is needed for achieving blocking^9^,^10^. In contrast, the prediction error theory predicts blocking with 1-trial XY training. One group of animals (blocking group) was subjected to 4-trial P+ training and subsequent 1-trial OP+ training. Another group (unpaired group) was subjected to unpaired presentation of a pattern and reward (P/+) 4 times each and then subjected to 1-trial OP+ training. As a prerequisite for this experiment, we showed that 1-trial OP+ training successfully leads to learning of the odor (W = 15, p = 0.0084, WCX test; compound group in Fig. 4a). The blocking group exhibited no significantly increased preference for the odor after training than that before training (W = 87, p = 0.76, WCX test; blocking group in Fig. 4a), whereas the unpaired group exhibited a significantly increased preference for the odor after training (W = 14, p = 0.0033, WCX test; unpaired group in Fig. 4a). Between-group comparison showed that the preference for the odor after training in the blocking group was significantly less than that in the compound group (U = 43.5, p = 0.00041) or the unpaired group (U = 269, p-value = 0.0096). The occurrence of blocking with 1-trial OP+ training rejected the attentional theory.

Alternatively, blocking with X+ training and subsequent 1-trial XY+ training might be due to a loss of attention to Y, which is presented together with X. This “naïve” attentional theory has not been seriously considered in mammals because it is thought to be too simplistic^10^, but this theory deserves consideration for blocking in crickets. In order to evaluate whether crickets' attention to an odor is lost after P+ training, their behavioral responses to an odor were observed. Crickets often extend and vibrate their maxillary palpi vigorously when they have perceived a food odor, which we refer to as maxillary palpi extension response (MER), but they do not exhibit MER when a visual pattern is presented. We compared the percentage of MER (%MER) to OP compound and that to a pattern alone to estimate crickets' response to the odor in the OP compound after P+ training. Two groups of animals were subjected to 4-trial P+ training and then, in one group, MER was tested to P alone and then to OP compound. In another group, the sequence of tests was reversed. Because the sequence of the tests had no effect on responses to OP compound or to P alone, the data from the two groups were pooled as a paired presentation group. The paired group exhibited a very low %MER to P alone but a high %MER to OP compound (χ^2^ = 9.3889, p = 0.0022, McNemar's test; Fig. 4b), suggesting that MERs to OP compound are, in most part, caused by the odor. Another group (unpaired group) was subjected to unpaired presentation of a pattern and reward (P/+) 4 times each, and then MERs to OP compound were tested. This group exhibited a high %MER to OP compound. The %MER to OP compound did not significantly differ between the P+ group and unpaired group (p = 0.43, Fisher's exact test). Assuming that MERs to OP compound are in large part due to the odor, this observation suggests that P+ training does not attenuate attention to the odor in OP compound. Thus, these observations rejected the naïve attentional theory.

### A neural circuit model of classical conditioning that matches prediction error theory

The results described above support the prediction error theory but do not unequivocally prove it, since the attentional theory is not the only alternative theory to account for blocking^6^,^7^,^8^. In order to obtain further evidence supporting the prediction error theory and rejecting alternative theories, we constructed a model of classical conditioning that matches the prediction error theory (Supplementary Fig. S1b) by modifying our previous model of classical conditioning in crickets^33^ (Supplementary Fig. S1a). An essential assumption in our new model is that pairing of CS and US leads to enhancement of synaptic transmission from “CS” neurons to three classes of neurons, i.e., “CR”, “OA1” and “OA2” neurons, in which “CS” neurons are neurons mediating CS, “CR” neurons are neurons whose activation leads to CR and “OA1” or “OA2” neurons are octopaminergic (OA) neurons mediating appetitive US and are inhibited or excited by activation of “CS” neurons, respectively. Other assumptions in the model and how this model matches the prediction error theory are described in legends of Supplementary Fig. S1. To better account for the model, information coded by “OA1” and “OA2” neurons is shown in Supplementary Table S1. We used this model for designing an experiment to test the prediction error theory.

### Demonstration of auto-blocking

We noticed that our model predicts that blockade of synaptic transmission from OA neurons by an OA receptor antagonist during a pairing of a stimulus (Y) with reward (Y+ training) impairs learning of Y but not formation of reward prediction by Y. This is because it impairs enhancement of “CS-CR” synapses but not that of “CS-OA1” and “CS-OA2” synapses in the circuitry, in which enhancement of all three types of synapses are necessary for achieving appetitive learning but that of “CS-OA1” synapses is sufficient for formation of reward prediction (see legends of Supplementary Fig. S1). Therefore, subsequent Y+ training, after recovery from the synaptic blockade caused by epinastine, should produce no learning. This effect can be termed “auto-blocking”, because learning of Y is blocked by US prediction by Y itself, not by X in the case of blocking. We used epinastine, an antagonist of the insect OA receptor^35^, which impairs appetitive learning but not aversive learning in crickets^30^,^31^.

**Figure 5:**
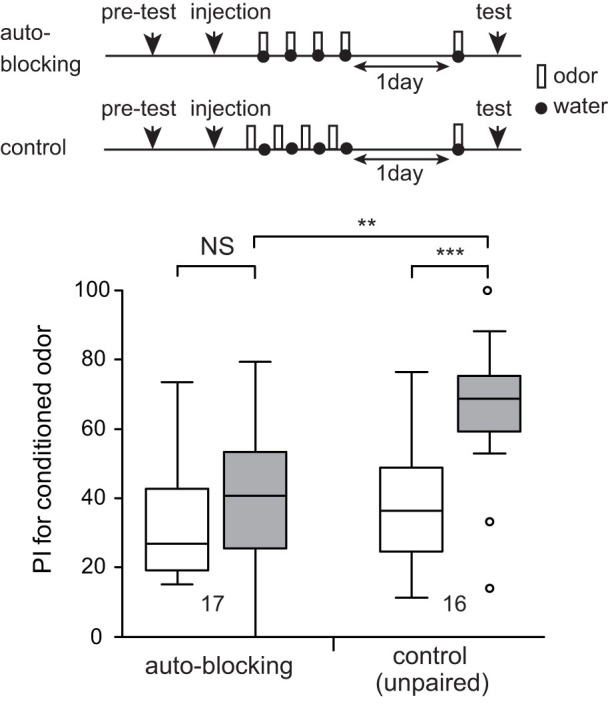
Auto-blocking. Two groups of animals were subjected to injection of 3 μl saline containing 2 μM epinastine. Thirty min later, one group (blocking group) was subjected to 4-trial pairing of an odor with water and another group (control group) was subjected to unpaired presentation of an odor and water 4 times each. The ITI was 5 min. On the next day, both groups were subjected to 1-trial pairing of the odor with water. Relative odor preference was tested before and at 30 min after training. PIs for the rewarded odor before and after training are shown as box and whisker diagrams. The number of animals is shown below the boxes. The WCX test was used for comparison of preferences before and after conditioning, and the M-W test was used to compare between groups. For multiple comparisons, Holm's method was used to adjust the significance level. The results of statistical comparison are shown as asterisks (*** p < 0.001; ** p < 0.01; NS p > 0.05).

We tested whether auto-blocking occurs in crickets. One group of animals (auto-blocking group) was injected with epinastine into the head haemolymph and the group was subjected to 4-trial O+ training 30 min later. The timing of injection and the concentration of the drug were based on our previous study^30^. The next day, the group was subjected to 1-trial O+ training. The group exhibited no significantly increased preference for the odor after training (W = 46, p = 0.27, WCX test; Fig. 5). Another group (unpaired group) was subjected to unpaired presentation of the odor and reward (O/+) 4 times each with application of epinastine and was subjected to 1-trial O+ training the next day. The unpaired group exhibited significantly increased preference for the odor after training (V = 5, p = 0.00061, WCX test). Between-group comparison showed that preference for the odor after training in the auto-blocking group was significantly less than that in the unpaired control group (U = 43, p = 0.0017, M-W test). The results demonstrate that auto-blocking occurs in crickets, providing evidence to further support applicability of prediction error theory to classical conditioning in crickets.

## Discussion

**Figure 6:**
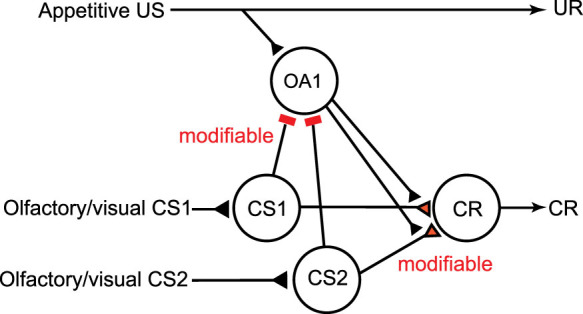
Accounts for blocking by our classical conditioning model. The model shown in Supplementary Fig. S1b assumes that pairing of a stimulus (CS1) with appetitive US leads to (1) an enhancement of inhibitory pathways from “CS1” neurons to “OA1” neurons and (2) that of excitatory synapses from “CS1” neurons to “CR” neurons, in which “CS1” are neurons mediating CS1, “OA1” are a type of octopaminergic (OA) neurons mediating appetitive US and “CR” are neurons whose activation leads to CR. During pairing of a compound of CS1 and CS2 with US after sufficient number of CS1-US pairing, “OA1” neurons are inhibited by activation of “CS1” neurons and thus responses of “OA1” neurons to US are diminished. As a result, no enhancement of “CS2-OA1” synapses and “CS2-CR” synapses occurs, in which “CS2” are neurons mediating CS2. Thus no learning of CS2 occurs. Synapses whose efficacy can be changed by conditioning are colored in red and marked as “modifiable”. Excitatory synapses are marked as triangles; inhibitory synapses are marked as bars. UR: unconditioned response. “OA2” neurons in our model are not illustrated in this figure for simplicity.

We obtained convincing evidence supporting the prediction error theory. At first, we obtained evidence of blocking, i.e., no learning of a stimulus (Y) by pairing of a compound of Y and another stimulus (X) with reward (XY+ training) when it is preceded by X+ training, in crickets. Among theories to account for blocking, we focused on prediction error theory^3^ and attentional theory^9^,^10^, the former accounting for blocking by lack of US prediction error and the latter by lack of attention to Y. The results of our experiment with 1-trial XY+ conditioning support the former theory but not the latter. In order to obtain further evidence for the prediction error theory, we constructed a neural circuitry model of classical conditioning that is consistent with the prediction error theory (Supplementary Fig. S1b), by revising our previous model^33^ (Supplementary Fig. S1a). How this model accounts for blocking is illustrated in Fig. 6. The model predicts that application of an OA receptor antagonist before Y+ training impairs learning of Y but not formation of reward prediction by Y (see legends of Supplementary Fig. S1 and Fig. 6). In accordance with this prediction, we observed no learning of Y by subsequent Y+ training. The finding of “auto-blocking” in crickets can be accounted for by the prediction error theory but not by other competitive theories to account for blocking (see below), providing rigorous evidence for validity of the prediction error theory.

Since predicting a future US from past experience may require sophisticated neural computation, some might argue that such computation is formidable for the small brains of insects. It should be noted, however, that our model suggests that computation of US prediction error can be achieved by a simple neural circuit consisting of a small number of elements.

It has remained controversial whether blocking occurs in learning of insects. Earlier studies using free-flight honey bees showed a blocking-like effect^17^,^18^, but in recent studies, the effect has been concluded to be due to confounding factors^23^,^24^. In olfactory conditioning of the proboscis extension response in harnessed honey bees, earlier studies also showed a blocking-like effect^19^,^20^,^21^, but in recent comprehensive studies, it was concluded that blocking is not a robust phenomenon^22^. Attempts to demonstrate blocking failed in the fruit-fly *Drosophila*^36^,^37^. These negative reports, however, do not necessarily indicate that blocking does not occur in these insects: Rather, they may indicate that more effort is needed to establish experimental paradigms to demonstrate blocking.

Suitable controls are needed to discriminate blocking from confounding factors, and there has been debate concerning the most appropriate control procedure to demonstrate blocking^23^,^28^. Considering the debate, we performed three different comparisons to demonstrate blocking, namely, we showed that (1) the blocking group exhibited no learning by comparing preferences for the CS before and after training, (2) preference for the CS after training in the blocking group was significantly less than that in the unpaired group, and (3) it was significantly less than that in the compound group. In the original experiment on blocking, Kamin^2^ used a group with XY+ compound conditioning as the control group, and this is still considered a typical control procedure. Some researchers, on the other hand, prefer to use unpaired presentation of X and US (+) before XY+ training in order to equalize the amount of exposure to X and US between the control group and the blocking group^16^. Other researchers argue that the use of between-group comparison is problematic and prefer within-group comparison^28^. Regardless of such debate, our study unequivocally demonstrates blocking in crickets.

Our model predicts “auto-blocking”, and we indeed observed this phenomenon. Importantly, our auto-blocking experiment demonstrated prediction error theory without using XY+ training and thus without stimulus competition between X and Y. To our knowledge, all theories other than the prediction error theory, including attentional theories^9^,^10^ and comparator hypothesis^11^, assume stimulus competition to account for blocking, and thus they fail to account for auto-blocking.

We propose that the auto-blocking procedure is applicable to learning systems of animals other than crickets. We noticed that auto-blocking can be predicted from computational models of the prediction error theory other than ours, such as a model proposed by Goel and Gelperin^38^. Their model differs in many respects from our model and thus does not account for learning in crickets, but it predicts that blockade of synaptic output from reinforcing neurons impairs learning but not formation of prediction error. Moreover, an auto-blocking experiment can be performed in any learning systems if the neurotransmitter of reinforcing neurons is known and synaptic output from reinforcing neurons can be blocked during learning. A combination of blocking and auto-blocking experiments should become a useful procedure for demonstration of the prediction error theory.

Although our model shown in Supplementary Fig. S1b focused on roles of OA neurons in conveying prediction error signals for appetitive US, we can similarly assume roles of dopamine (DA) neurons in conveying prediction error signals for aversive US, and confirmation of these is one of our major goals. We hypothesize that OA and DA neurons projecting to the lobes of the mushroom body convey prediction error signals for olfactory conditioning, because the lobes have been suggested to be the sites of association between CS and US^14^,^39^. It would be interesting to compare activities of these neurons in insects to those of midbrain DA neurons in mammals, which have been suggested to convey prediction error signals for classical conditioning^1^,^4^ and instrumental conditioning^5^.

Neural circuitry mechanisms for computation of the prediction error remain unknown in any learning systems, and crickets should emerge as pertinent models in which to elucidate this important subject. Our model predicts that (1) there should be two types of OA or DA neurons, one type being inhibited and the other type being excited by CS presentation after conditioning and (2) the former conveys US prediction error and activation of them is needed for enhancement of one type of synapses, whereas activation of the latter is needed for memory retrieval. These predictions should guide our future electrophysiological studies. It should also be noted that our model indicates that the prediction error theory does not account for all aspects of associative learning in crickets. The model assumes synaptic plasticity in three different synapses in the circuitry and suggests that the plasticity of one type of synapses (“CS-CR” synapses) is governed by US prediction error but that of other synapses (“CS-OA1” synapses and “CS-OA2” synapses) is governed by US (see legends of Supplementary Fig. S1). Therefore, actual mechanisms of associative learning in insects may be more elaborate than the prediction error theory assumes. This argument may be applicable to mammals: Because the prediction error theory is known to account for some but not all features of associative learning in mammals^6^,^7^,^8^, further effort is needed to delineate the extent by which the prediction error theory accounts for mechanisms of associative learning in mammals.

## Methods

### Insects

Adult male crickets, *Gryllus bimaculatus*, at 1 week after the imaginal molt were used. Before the experiment, animals were placed individually in beakers and deprived of drinking water for 4 days to enhance their motivation to search water.

### Olfactory and Visual Conditioning Procedure

We used classical conditioning and operant testing procedures described previously (Fig. 1)^30^,^31^,^40^. In olfactory conditioning, peppermint odor was used as conditioned stimulus (CS), while in visual conditioning, a white-center and black-surround pattern (white-center pattern) was used as CS. In compound conditioning, the odor and pattern were presented simultaneously (compound CS). Water was used as US (reward). A syringe containing water was used to present CS and US to each cricket. A filter paper soaked with peppermint essence or a white-center pattern was attached to the needle of the syringe (Fig. 1). For pairing trial, an odor and/or visual pattern was approached to the antennae or the head and held for 3 sec, and subsequently a drop of water was attached to its mouth. Crickets typically received 4-trial training with an inter-trial interval (ITI) of 5 min. After olfactory or compound conditioning trials, the air in the beaker was ventilated.

### Preference Tests

The odor and pattern preference tests were carried out as described elsewhere^30^,^31^,^40^. All groups were tested with relative preference between the conditioned (peppermint) and control (vanilla) odor or between the conditioned (white-center) and control (black-center) pattern before and at 30 min after conditioning. In the chamber to test odor preference, the floor had two holes that connected the chamber with two cylindrical odor sources containing a filter paper soaked with either peppermint or vanilla essence and covered with fine gauze net (Fig. 1). Three containers were mounted on a rotative holder and two of the three sources could be located simultaneously beneath the holes of the test chamber. In the apparatus for the pattern preference test, two white-center patterns and one black-center pattern were displayed on a gray sliding wall at the end of the test chamber, and a white-center pattern and a black-center pattern could be presented at the same time (Fig. 1). Before testing, a cricket was transferred to the waiting chamber and left for about 4 min to become accustomed to the surroundings. Then the cricket was allowed to enter the test chamber and then test started. Two min after the test start, the relative positions of the odor sources or patterns were changed by either rotating the container holder or sliding the wall. The preference test lasted for 4 min. If the total visiting time of a cricket to odor sources or patterns was less than 10 s, we considered that the animal was less motivated, possibly due to a poor physical condition, and the data were rejected. Animals that fell into this category comprised less than 10% of the total.

We measured maxillary palpi extension response (MER) to observe crickets' attention to an odor in the odor-pattern compound (see Fig. 1). Crickets often extend and shake their maxillary palpi vigorously when a small filter paper soaked with an essence of a food-related odor is approached to their antennae or when water or sucrose solution has been attached to the mouth or antennae. We recorded MER if a cricket extended its maxillary palpi during 3-sec period in which a small filter paper soaked with odor essence was presented within 1 cm of the antennae.

### Pharmacology

Animals were injected with 3 μl of saline containing 2 μM epinastine (Sigma-Aldrich, Tokyo) into the head hemolymph. The estimated final concentration after circulation is 7.0 nM^30^.

### Statistical Analysis

In the test, we considered that an odor or pattern was visited when the cricket probed it with its mouth or palpi. The time visiting each odor or pattern was measured cumulatively. Relative preference of each animal was determined using the preference index (PI) for rewarded odor or pattern, defined as *t_r_*/(*t_r_*+*t_nr_*) × 100, where *t_r_* was the time spent exploring the odor or pattern associated with reward and *t_nr_* was the time spent exploring the odor or pattern not associated with reward. Wilcoxon's test (WCX test) was used to compare preferences before and after training. The Mann-Whitney test (M-W test) was used to compare preferences of different groups. Holm's sequential Bonferroni procedure (Holm's method) was used to adjust the p-values for multiple comparisons. We found no significantly different odor or visual pattern preferences among the different groups of animals before training (Kruskal-Wallis test, p > 0.6).

The percentage of MER was calculated as the number of crickets that showed MER to an odor or pattern with respect to the total number of crickets tested. The difference in response level to the CS was evaluated by means of a McNemar test. Differences in CS responses between groups were assessed using Fisher's exact tests.

## Author Contributions

Conceived and designed the experiments: K.T., Y.M. and M.M. Performed the experiments: K.T. Analyzed the data: K.T. and M.M. Wrote the manuscript: K.T., Y.M. and M.M.

## Supplementary Material

Supplementary Information Supplementary materials

## Figures and Tables

**Table 1:** Procedures and results of blocking experiment

Group	Phase 1 training	Phase 2 training	Results: Learning of Y?	Figures
Compound	-	XY+	Yes	Figs. 2, 4
Blocking	X+	XY+	No	Figs. 3, 4
Unpaired (control for blocking)	X/+	XY+	Yes	Figs. 3, 4
Auto-blocking	Y+ (under epinastine)	Y+	No	Fig. 5
Unpaired (control for auto-blocking)	Y/+ (under epinastine)	Y+	Yes	Fig. 5

XY+: a compound of stimulus X and stimulus Y is paired with appetitive US; (+): reward; Y/+: unpaired presentation of stimulus Y and reward. In most experiments, X is a visual pattern (P) and Y is an odor (O) (Fig. 1a); in other experiment, the stimulus arrangement is reversed (Fig. 1b).
