# Supplementary material for: Critical evidence for the prediction error theory in associative learning
Source: Sci Rep. 2015 Mar 10;5:8929. doi: 10.1038/srep08929 (PMC4354000; doi:10.1038/srep08929)
Supplement: Supplementary Information — Supplementary materials [file srep08929-s1.pdf]

Title: Critical evidence for the prediction error theory in associative learning

Authors: Kanta Terao, Yukihisa Matsumoto, Makoto Mizunami

Supplemenatary Figure S1.

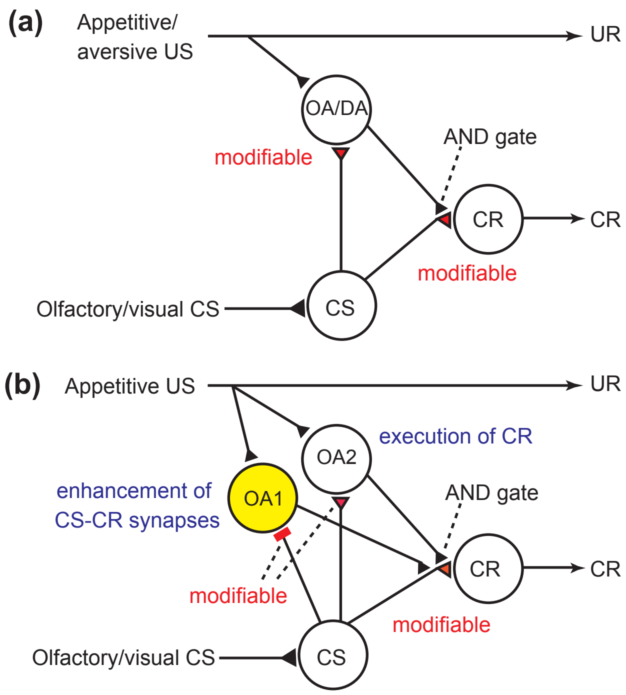

Supplementary Figure S1. Models of classical conditioning in crickets. (a) A model of appetitive conditioning, which we proposed to account for our finding that OA or DA receptor antagonists impair learning and execution of conditioned response (or memory retrieval) in appetitive or aversive conditioning, respectively[1]. The model assumes that (1) "CS" neurons (which may represent intrinsic neurons of the mushroom body) that convey signals about CS make silent or weak synaptic connections with dendrites of "CR" neurons (which may represent efferent (output) neurons of the mushroom body lobe), activation of which leads to a conditioned response (CR), but these synaptic connections are silent or very weak before conditioning, (2) OA or DA neurons ("OA/DA" neurons), which convey signals for appetitive or aversive US, respectively, make synaptic connections with axon terminals of "CS" neurons, (3) "CS" neurons also make silent synaptic connection with "OA/DA" neurons (which might not be monosynaptic), (4) the efficacy of the synaptic transmission from "CS" neurons to "CR" neurons and to "OA/DA" neurons is strengthened by coincident activation of "CS" neurons and "OA/DA" neurons during appetitive or aversive conditioning and (5) after conditioning, activation of "CS" neurons activates "OA" neurons and coincident activation of "CS" neurons and "OA/DA" neurons is needed for activation of "CR" neurons (AND gate) and for production of conditioned responses to CS. UR: unconditioned response. (b) A new model of the roles of OA neurons in appetitive conditioning to match the prediction error theory. In the model, we assumed "OA1" neurons (colored in yellow) that govern enhancement of "CS-CR" synapses (but not execution of CR), in addition to "OA2" neurons that govern execution of CR or memory retrieval (but not enhancement of "CS-CR" synapses). OA2 neurons but not OA1 neurons govern the "AND gate". The "OA1" neurons are assumed to receive silent or very weak inhibitory synapses from "CS" neurons before training, which are strengthened by CS-US pairing. During training, "OA1" neurons receive excitatory synaptic input representing actual US and inhibitory input from "CS" neurons representing US predicted by CS, and thus their activities represent US prediction errors, thereby allowing US prediction error signals to govern enhancement of synaptic transmission.

1. Mizunami, M., *et al.* Roles of octopaminergic and dopaminergic neurons in appetitive and aversive memory recall in an insect. *BMC Biol.* **7**, 46 (2009).

Supplementary Table S1. Information coded in the responses of OA1 and OA2 neurons in our model.

Stimulus	OA1		OA2	
	Before training	After training	Before training	After training
US	1(US)	1 (US)	1(US)	1 (US)
CS	0	0 [-1 (-USP)]*	0	1 (USP)
CS+US	1 (US)	0 (USPE)	1 (US)	1(US+USP)

Responses of OA1 and OA2 neurons in the model shown in Fig. 5b to US (reward), CS, and paired presentation of CS and US before and after conditioning. After completion of training, OA1 neurons that govern enhancement of synaptic transmission underlying conditioning exhibit no responses to paired CS-US presentation, and thus no further enhancement of synaptic transmission occurs. USP: US prediction; USPE: US prediction error. Responses are indicated as all or none (1 or 0). *Negative value in the parentheses indicates inhibitory synaptic input.
